# Glucose metabolic reprogramming: a novel strategy to enhance radiotherapy response to triple-negative breast cancer

**DOI:** 10.3389/fpubh.2026.1715716

**Published:** 2026-04-21

**Authors:** Lu Gan, Qian Li, Boyi Yu, Jing Si, Qiang Li, Weiqiang Chen, Bing Wang

**Affiliations:** 1 Institute of Modern Physics, Chinese Academy of Sciences, Lanzhou, China; 2 Key Laboratory of Heavy Ion Radiation Biology and Medicine, Institute of Modern Physics, Chinese Academy of Sciences, Lanzhou, China; 3 Key Laboratory of Basic Research on Heavy Ion Radiation Application in Medicine, Institute of Modern Physics, Chinese Academy of Sciences, Lanzhou, Gansu, China; 4 College of Nuclear Science and Technology, University of Chinese Academy of Sciences, Beijing, China; 5 Hospital of Stomatology, Lanzhou University, Lanzhou, China; 6 Institute for Radiological Science, National Institutes for Quantum Science and Technology (QST), Chiba, Japan

**Keywords:** biomarker, glucose metabolic reprogramming, radiosensitivity, radiotherapy, triple-negative breast cancer

## Abstract

Triple-negative breast cancer (TNBC) is an aggressive subtype of breast cancer characterized by poor prognosis and limited responsiveness to conventional therapies. Increasing evidence shows that the reprogramming of glucose metabolism is a hallmark of cancer cells, supporting their rapid proliferation, metastatic potential, and therapy resistance. This metabolic shift is particularly pronounced in TNBC, where reliance on glycolysis is greater than in other breast cancer subtypes. Consequently, strategies that target glucose metabolic pathways may offer a promising means to overcome treatment resistance and improve clinical outcomes. In this review, we summarize the unique features and regulatory mechanisms of glycolytic reprogramming in TNBC, with attention to tumor heterogeneity and its implications for disease progression and treatment response. We highlight recent preclinical studies that evaluate therapeutic approaches designed to exploit metabolic vulnerabilities, including glycolysis inhibition, metabolic enzyme targeting, and combination regimens with radiotherapy. Collectively, these findings suggest that interventions aimed at glycolytic pathways hold considerable potential to enhance radiosensitivity in TNBC. We discuss the translational prospects of this research, emphasizing the value of glycolysis-related genes as predictive biomarkers and as foundations for the development of novel targeted agents. While preliminary evidence is encouraging, further validation is required to establish the safety, efficacy, and clinical applicability of these strategies in human patients. Continued research in this area is expected to contribute to the development of more effective therapeutic options, ultimately improving the management and prognosis of TNBC.

## 1 Introduction

Breast cancer remains the most frequently diagnosed cancer among women worldwide and ranks as the second leading cause of cancer-related mortality (1). Within its heterogeneous subtypes, triple-negative breast cancer (TNBC) accounts for approximately 15%−20% of cases and is characterized by the absence of estrogen receptor, progesterone receptor, and human epidermal growth factor receptor 2 (HER2) expression. TNBC is particularly aggressive, with a higher likelihood of early recurrence, distant metastasis, and reduced 5-year survival compared with hormone receptor–positive or HER2-positive breast cancers (2). Due to the lack of specific therapeutic targets, TNBC patients rely mainly on surgery, chemotherapy, and radiotherapy, yet the prognosis remains poor, underscoring the urgent need for new therapeutic strategies (3–5).

One of the defining features of TNBC biology is metabolic reprogramming, a hallmark of cancer that supports tumor growth, invasion, and therapeutic resistance (6). Among these alterations, glucose metabolism plays a pivotal role. Unlike normal cells, which preferentially utilize mitochondrial oxidative phosphorylation under aerobic conditions, TNBC cells often adopt the Warburg effect, favoring glycolysis even in the presence of sufficient oxygen (7). This shift enables rapid ATP generation and provides biosynthetic intermediates such as nucleotides, amino acids, and lipids, thereby fueling the malignant behaviors of TNBC. Furthermore, TNBC displays an even greater dependence on glycolysis than other breast cancer subtypes, which enhances its capacity for proliferation, migration, and immune evasion (8, 9). These insights not only deepen our understanding of TNBC biology but also reveal opportunities for therapeutic intervention. Targeting key enzymes, transporters, or regulatory pathways involved in glucose metabolism may not only suppress TNBC progression but also improve prognosis through metabolic precision therapy.

Radiotherapy remains an integral component of TNBC treatment, improving local control and survival outcomes (10). However, despite advances in modern radiation delivery techniques such as intensity-modulated radiotherapy and proton therapy, locoregional recurrence continues to occur in a significant proportion of patients (11). This therapeutic challenge is often attributed to the survival of intrinsically resistant tumor subsets or the emergence of radiation-tolerant clones following treatment. Radiosensitivity refers to the degree to which cells, tissues, organs or organisms response to ionizing radiation (12). The primary biological determinant of radiotherapy failure is the radioresistance to radiotherapy (13). Tumor cells with high radiosensitivity are more likely to undergo cell-cycle arrest or apoptosis following irradiation, thereby contributing to a favorable therapeutic response, whereas low sensitivity (i.e., radioresistance) results in inadequate tumor control (14). Radiosensitivity varies among different tumor cell populations, and is influenced by genetic factors as well as the complexity and heterogeneity of underlying signaling pathways, all of which can modulate cellular response to radiation (15). In TNBC, radiation resistance raises from the convergence of multiple molecular mechanisms, including dysregulated DNA damage response signaling, immunosuppressive tumor microenvironment remodeling, and metabolic reprogramming (16). In particular, glycolysis-driven metabolic alterations promote radioresistance by enhancing pro-survival signaling, facilitating DNA repair, and enabling immune evasion, thereby reducing radiosensitivity and ultimately compromising radiotherapy efficacy.

In this review, we focus on the distinct features of glucose metabolic reprogramming in TNBC and examine its role in shaping radiosensitivity. By elucidating the links between glycolysis, tumor progression, and radiotherapy outcomes, we aim to provide a framework for targeting metabolic vulnerabilities as a means of overcoming radioresistance in TNBC.

## 2 Glucose metabolism reprogramming in TNBC

In recent years, considerable progress has been achieved in elucidating the mechanisms of glucose metabolic reprogramming in TNBC. Distinct metabolic features of TNBC not only illuminate its highly aggressive biological behavior but also provide potential therapeutic targets. TNBC is characterized by a predominantly glycolytic phenotype, marked by the diversion of glycolytic intermediates into anabolic pathways and mitochondrial dysregulation, hallmarks that contribute to tumor aggressiveness and metabolic flexibility (17, 18). Altered glucose metabolism in TNBC manifests through enhanced glucose uptake, hyperactivated glycolysis, reduced oxidative phosphorylation (OXPHOS), and the accumulation of lactate (19).

Interestingly, TNBC does not adhere strictly to the traditional Warburg effect of suppressed mitochondrial respiration. Instead, some TNBC tumors exhibit a dual metabolic phenotype, simultaneously engaging glycolysis and OXPHOS. This hybrid state, rather than sole reliance on glycolysis, plays a pivotal role in tumor aggressiveness, metastatic potential, and therapy resistance, thereby emphasizing the need to identify oncogenes and signaling pathways that promote elevated OXPHOS activity (20, 21). Enhanced aerobic glycolysis in TNBC not only generates ATP but also supplies critical biosynthetic precursors, including nucleotides, amino acids, and lipids, that sustain tumor growth and enable adaptation under stressful conditions such as hypoxia, detachment-induced anoikis, or therapy-induced DNA damage (22).

### 2.1 Characteristics of glucose metabolic reprogramming in TNBC

#### 2.1.1 Abnormal glucose uptake

Glucose transporters (GLUTs) are essential regulators of cellular glucose metabolism and play central roles in TNBC biology. Fluorodeoxyglucose ([^18^F]-FDG) is the most widely used positron emission tomography (PET) tracer in clinical practice, based on the increased glucose uptake characteristic of malignant cells. This property enables both lesion localization and assessment of metabolic activity. Owing to metabolic heterogeneity among breast cancer subtypes, [^18^F]-FDG uptake varies considerably, with TNBC generally exhibiting higher uptake than non-TNBC tumors. This is largely attributed to the high proliferative activity of TNBC cells and the overexpression of GLUTs, resulting in markedly increased [^18^F]-FDG accumulation (23). The androgen receptor (AR) plays an important role in the development and progression of breast cancers. Several studies have demonstrated that AR-negative TNBC is frequently associated with poorer clinical outcomes (24–26). Accordingly, therapeutic strategies targeting AR signaling have been explored in TNBC, leading to clinical investigations of AR-directed agents such as enzalutamide and seviteronel (27, 28). In addition, increased [^18^F]-FDG uptake has been associated with aggressive tumor phenotypes and treatment responsiveness, including reported sensitivity to AR-targeted therapies and immunotherapy (29). [^18^F]-FDG PET/CT also plays a particularly important role in disease staging, especially in patients with stage II–III TNBC (30). TNBC is a highly heterogeneous disease, and this heterogeneity contributes substantially to its malignant potential. Metastatic lesions may differ markedly from the primary tumor, and from each other, in both biological characteristics and metabolic profiles. Consequently, metabolic insights derived from primary tumors may not always be applicable to metastatic disease. Consistent with this notion, patients with metastatic TNBC exhibiting a high heterogeneity index (HI) and high maximum FDG uptake (MAX) have been reported to experience shorter progression-free survival, with elevated MAX values also associated with poorer prognosis (31). With the rapid development of nuclear medicine, the application scope of radioactive drugs is also constantly expanding. Identifying prostate-specific membrane antigen (PSMA) as a target for radionuclide diagnosis and treatment of prostate cancer has become a clinical breakthrough in nuclear medicine. In a prospective study comparing [^18^F]-PSMA-1007 and [^18^F]-FDG PET/CT for the detection of metastatic lesions in TNBC, [^18^F]-PSMA-1007 demonstrated higher maximum standardized uptake values (SUVmax) in distant metastases (32), suggesting potential complementary roles for alternative tracers. TNBC is also characterized by high Ki-67 expression, frequent p53 mutations, and high nuclear grade, all of which are associated with unfavorable clinical outcomes. However, it should be noted that FDG uptake may yield false-positive results following surgery, biopsy, or radiotherapy, potentially compromising diagnostic accuracy (33). Accordingly, combined assessment using FDG-PET/CT together with molecular biomarkers such as Ki-67, p53, and EGFR has been proposed to improve diagnostic precision and therapeutic decision-making in TNBC (34). Nevertheless, the clinical translation of emerging imaging targets and biomarkers requires validation in larger, well-designed prospective studies. In particular, GLUT1 and GLUT3 are highly expressed in TNBC and are strongly linked to malignant traits. GLUT1 overexpression facilitates enhanced glucose uptake, directly fueling cell proliferation, invasion, and metastatic spread (35). Silencing GLUT1 suppresses proliferation (36), and clinical data confirm that elevated GLUT1 expression correlates with poor prognosis, greater recurrence, and higher metastatic risk in TNBC patients (37). GLUT3, which has high affinity for glucose, ensures sufficient uptake even in low-glucose environments, thereby supporting cell growth, migration, and survival under metabolic stress (38).

In addition to GLUT1 and GLUT3, GLUT4, an insulin-responsive transporter, also contributes to TNBC metabolism. Downregulation of GLUT4 expression or activity reduces glucose uptake and lactate production, prompting a metabolic shift away from glycolysis toward OXPHOS. This shift attenuates proliferation and compromises TNBC survival, particularly under hypoxic conditions (39). Beyond GLUTs, other molecular regulators, including amino acid transporters and cooperative proteins, appear to modulate glucose uptake in TNBC cells (40, 41). Although the precise mechanisms remain to be fully elucidated, these molecules likely participate in metabolic crosstalk, influencing tumor growth and survival. Future studies should clarify their functional roles and interactions with GLUTs, which may uncover novel therapeutic opportunities for TNBC.

#### 2.1.2 Changes in glucose metabolic pathways

##### 2.1.2.1 Enhancement of glycolysis

Compared with other breast cancer subtypes, TNBC cells are more reliant on glycolysis, exhibiting elevated glucose uptake and lactate secretion (42). This glycolytic preference is mediated by aberrant regulation of key enzymes, such as hexokinase (HK), phosphofructokinase (PFK), and pyruvate kinase (PK), which together exert profound effects on energy production and metabolite synthesis (43).

HK catalyzes the phosphorylation of glucose to glucose-6-phosphate, a crucial first step that traps glucose within the cell. Among its isoforms, HK2 is markedly upregulated in TNBC compared with normal breast tissue, and this elevated expression correlates with tumor proliferation, invasion, and metastasis (44, 45). Total and particulate HK activity is significantly high in TNBC compared to other type breast cancers (46, 47). Studies have demonstrated that the glycolysis inhibitor 3-bromopyruvate suppresses the growth of TNBC in mouse models by reducing HK activity, down-regulating c-Myc expression, and decreasing glycolytic energy production (48). In addition, a cancer metabolic reprogramming-enabling photoresponsive nanoproteolysis-targeting chimera has been shown to inhibit the proliferation of TNBC cells *in vitro* through targeted degradation of HK2 (49).

PFK-1 catalyzes the conversion of fructose-6-phosphate to fructose-1,6-bisphosphate, a key regulatory step in glycolysis (50). Among its isoforms, the platelet-type PFK (PFKP) is significantly upregulated in TNBC tissues and cell lines. Elevated PFKP expression promotes tumor proliferation, invasion, and migration, whereas silencing PFKP suppresses these malignant behaviors (51, 52). Yang et al. (53) demonstrated that coenzyme Q0 suppresses the growth of TNBC cells under hypoxic conditions by downregulating the expression of HIF-1α, GLUT1, and key glycolysis-related proteins, including HK-2, LDH-A, and PFK-1.

PK catalyzes the final step of glycolysis, converting phosphoenolpyruvate (PEP) into pyruvate and ATP. Cancer cells predominantly express the M2 isoform (PKM2), in contrast to the M1 isoform (PKM1) found in normal cells (54). PKM2 is significantly upregulated in TNBC, and its expression correlates with larger tumor size, lymph node metastasis, and poor overall survival. Patients with lower PKM2 levels tend to have better prognoses, while PKM2 knockdown impairs TNBC proliferation and metastasis (55). PKM2 not only drives glycolytic flux but also serves as a cancer biomarker and regulator of oncogenic pathways. PKM2 phosphorylation marks aggressive breast cancer cell phenotypes. Studies have shown that in TNBC cells, the phosphorylation of PKM2 at the S37 site is associated with the cyclin-dependent kinase (CDK) pathway. In a TNBC mouse xenograft model, treatment with TEPP-46 or the potent CDK inhibitor dinaciclib can both inhibit tumor growth and reduce PKM2pS37 levels. The combination of the two drugs can also reduce cell invasion, disrupt redox balance, and induce cancer cell death (56). Therapeutic strategies targeting PKM2, including inhibitors such as shikonin or co-delivery nanoparticle systems combining paclitaxel, show promise in suppressing epithelial–mesenchymal transition (EMT), metastasis, drug resistance, and tumor growth (57). Additionally, PKM2 has been implicated in radiation response; preclinical studies suggest that combining PKM2 modulators with radiotherapy may sensitize TNBC to radiation and improve therapeutic outcomes (58).

##### 2.1.2.2 Alteration in oxidative phosphorylation (OXPHOS)

Cancer cells exhibit remarkable metabolic plasticity, dynamically reprogramming their metabolic networks to adapt to environmental stressors, such as nutrient deprivation, chemotherapy, and metastatic dissemination (17, 59, 60). Compared with normal breast tissue, OXPHOS is among the most significantly upregulated metabolic pathways in TNBC (61). Moreover, chemotherapy-resistant TNBC cells preferentially rely on OXPHOS for energy production compared with chemotherapy-sensitive cells (62). Notably, TNBC stem-like cells exhibited elevated OXPHOS activity, which is associated with enhanced chemoresistance (63). Under these conditions, increased reliance on OXPHOS allows cells to sustain ATP production and maintain redox balance, thereby facilitating survival, therapy resistance, and progression (64). This makes OXPHOS a critical metabolic vulnerability in TNBC.

Mitochondria are the primary site of oxidative metabolism, where energy is generated through the oxidation of glucose, fatty acids, and amino acids. They also represent the major sources of intracellular ROS production. Targeting mitochondrial respiration and metabolic pathways in tumor cells has emerged as an effective strategy to enhance the efficacy of radiotherapy across multiple cancer types (65). Disruption of mitochondrial flux or interference with the expression or function of proteins encoded by mitochondrial DNA (mtDNA) can impair mitochondrial capacity, leading to altered energy metabolism and reduced cellular resilience to radiation-induced stress (66). In this context, elevated expression of Elongin B (ELOB) has been associated with poor prognosis in TNBC. Notably, genetic ablation of ELOB in TNBC cells leads to reduced mtDNA expression, impaired mitochondrial respiratory function, and a concomitant increase in radiosensitivity (67). In subsets of TNBC cells, enhanced pyruvate entry into mitochondria leads to altered tricarboxylic acid (TCA) cycle flux and increased reliance on OXPHOS. For example, glutamine supplementation augments TCA intermediates, including succinate, fumarate, and malate, in a dose-dependent manner (68). The leucine-rich pentatricopeptide repeat-containing protein (LRPPRC) is markedly overexpressed in TNBC and promotes OXPHOS by upregulating mitochondrial-encoded OXPHOS subunits; its knockdown reduces OXPHOS activity and correlates with improved patient prognosis (69). Similarly, TMEM65, a mitochondrial inner-membrane protein, enhances OXPHOS by activating the OXPHOS–ROS–HIF-1α signaling axis and transactivating SERPINB3, thereby driving tumor growth, metastasis, and cisplatin resistance (61). In addition, monocarboxylate transporter 1 (MCT1) is a marker of mitochondrial metabolism, which is involved in the transport of lactic acid to promote metabolic reprogramming during tumor progression (70). MCT1 inhibitors can effectively inhibit the proliferation of TNBC cells both *in vivo* and *in vitro* (71). Collectively, these findings underscore mitochondrial reprogramming as a central driver of TNBC aggressiveness and therapeutic resistance, highlighting mitochondria-targeted strategies as potential interventions.

##### 2.1.2.3 Alterations in the pentose phosphate pathway (PPP)

The pentose phosphate pathway (PPP), a major metabolic branch of glycolysis, fulfills anabolic demands by supplying NADPH for redox homeostasis and ribulose-5-phosphate for nucleotide synthesis. The oxidative arm of the PPP generates NADPH to maintain cellular redox balance, whereas the non-oxidative arm produces fructose-6-phosphate (F6P) and glyceraldehyde-3-phosphate (G3P), which are recycled into glycolysis (72). In TNBC, this metabolic reprogramming is not merely adaptive; by providing reducing equivalents and biosynthetic precursors, the PPP supports nucleic acid and fatty acid synthesis and protects tumor cells from oxidative stress induced by chemotherapy and radiotherapy, two key mechanisms underlying treatment resistance (73). Consistent with these findings, PPP-dependent stress resistance has been reported in other aggressive malignancies, including non-small cell lung cancer and colorectal cancer, where PPP activation correlates with enhanced survival under therapeutic pressure (74–76).

Glucose-6-phosphate dehydrogenase (G6PD), the rate-limiting enzyme of the oxidative arm of the PPP, is frequently upregulated in TNBC. Elevated G6PD expression is associated with poor prognosis, whereas G6PD silencing activates AMP-activated protein kinase (AMPK) signaling, leading to suppressed tumor cell proliferation and survival (77–79). Importantly, the PPP/G6PD axis has emerged as a promising therapeutic target. Preclinical studies demonstrate that G6PD inhibitor dehydroepiandrosterone (DHEA) sensitizes TNBC cells to chemotherapy (80). Although G6PD-targeted agents have not yet advanced to late-phase clinical trials, early preclinical evaluations of novel inhibitors, such as G6PDi-1, show improved specificity and reduced off-target toxicity, thereby providing a foundation for future clinical translation (81). Collectively, these findings identify the PPP as a conserved hallmark of metabolic reprogramming in TNBC and other aggressive cancers, with substantial potential for therapeutic exploitation to overcome treatment resistance, and position G6PD as a promising prognostic biomarker.

The above paragraphs describe the key regulatory factors that affect the occurrence and development of TNBC. Intervention targeting these targets provides new potential possibilities for improving the therapeutic efficacy of TNBC. At present, relatively few drugs targeting glucose metabolism reprogramming in TNBC have entered the clinical trial stage, and the relevant drugs targeting this target and their clinical trial progress are listed in Table 1.

**Table 1:** Clinical trials of drugs targeting glucose metabolism reprogramming in TNBC.

Drugs	Target/mechanism	Clinical trial	Trial
AZD3965	Monocarboxylate transporter 1 (MCT1). Inhibit the excretion of lactic acid	Phase I	NCT01791595 (clinicaltrials.gov)
Dichloroacetate (DCA)	PDK	Phase II	NCT01029925 (clinicaltrials.gov)
Alkaline glucosodiene molecules	Glucose	Phase I	NCT05957939 (clinicaltrials.gov)
Metformin	Glucose	Phase II	CTRI/2022/05/042576 (Clinical Trials Registry of India)
CB-839	Glutaminase. Inhibit the glutamine decomposition pathway and induce cellular energy metabolism reprogramming	Phase II	NCT03057600 (clinicaltrials.gov)
Combination of veliparib + lapatinib	Targeted PARP and ErbB-2/EGFR	Phase II	NCT02158507 (clinicaltrials.gov)

## 3 Factors affecting sensitivity to radiotherapy related to glycolysis in TNBC

TNBC is characterized by pronounced glycolytic reprogramming, which not only sustains rapid tumor proliferation but also confers substantial resistance to radiotherapy through modulation of cellular redox homeostasis, DNA repair capacity, and stress response pathways. Among the numerous regulators of glycolytic metabolism and oncogenic signaling, c-Myc/TXNIP, HIF-1, p53, and PTEN were specifically selected based on three core rationales. First, these regulators function as critical nodes that integrate glycolytic flux with key oncogenic signaling networks, thereby acting as “bridge molecules” linking metabolic adaptation to malignant progression in TNBC. Second, accumulating preclinical and clinical evidence directly implicates dysregulation of these pathways in radiotherapy resistance, underscoring their clinical relevance as potential therapeutic targets. Third, these regulators encompass distinct yet complementary dimensions of glycolysis-associated radiosensitivity, providing a comprehensive framework for dissecting the multilayered mechanisms underlying treatment resistance. The factors discussed below illustrate the intricate interplay between glycolytic metabolism and oncogenic signaling in TNBC. Elucidating these mechanisms not only deepens our understanding of the metabolic vulnerabilities that drive therapeutic resistance but also offers potential strategies to enhance radiosensitivity and ultimately improve patient outcomes.

### 3.1 c-Myc and Thioredoxin-interacting protein (TXNIP)

The transcription factor c-Myc exerts profound influence over diverse cellular processes, including proliferation, differentiation, apoptosis, and metabolism. Dysregulation of c-Myc, whether by mutation or overexpression, has long been associated with tumor initiation and progression. One key mechanism is its ability to reprogram cellular metabolism by driving glycolysis, a hallmark of the Warburg effect (82). Numerous glycolytic genes are direct transcriptional targets of c-Myc, including GLUT1, phosphoglucose isomerase, phosphofructokinase, glyceraldehyde-3-phosphate dehydrogenase, phosphoglycerate kinase, and enolase (83).

In TNBC, both c-Myc and GLUT1 are highly expressed. GLUT1 facilitates glucose uptake across the plasma membrane, while HK, another c-Myc-regulated enzyme, catalyzes the first committed step of glycolysis. The concerted upregulation of GLUT1 and HK enhances aerobic glycolysis, thereby supporting tumor growth and survival (84). Recent evidence demonstrates that 3-bromopyruvic acid can disrupt this pathway by targeting the c-Myc/TXNIP axis, ultimately triggering mitochondria-mediated apoptosis in TNBC cells (85). Similarly, β-Escin reduces glutamine metabolism by downregulating c-Myc, attenuating the aggressive phenotype of MDA-MB-231 cells (86). Furthermore, the interaction between METTL3 and the c-Myc/WDR5 complex has been implicated in promoting glycolysis, underscoring the multifaceted role of c-Myc in metabolic adaptation (87).

TXNIP, also known as thioredoxin-binding protein-2, is a ubiquitously expressed regulator that inhibits thioredoxin (TXN) function. mRNA and single-cell RNA analyses have identified TXNIP as a mediator that enhances antitumor immune responses in TNBC, suggesting its utility as both a biomarker and therapeutic target (88). Functionally, TXNIP suppresses glycolysis, acting in opposition to c-Myc. The EGFR–MYC–TXNIP axis has emerged as a central pathway in TNBC metabolism, with pharmacologic agents such as silibinin showing promise in targeting this regulatory circuit (89). Importantly, a Myc (high)/TXNIP (low) gene signature correlates with poorer survival in TNBC patients, reflecting a distinct metabolic phenotype not observed in non-TNBC breast cancers (90).

### 3.2 HIF-1α

Hypoxia-inducible factor-1α (HIF-1α) is a master transcriptional regulator activated under hypoxic conditions, a common feature of the tumor microenvironment. In breast cancer, elevated HIF-1α is linked to tumor aggressiveness, therapeutic resistance, and poor prognosis (91). Functionally, HIF-1α promotes glycolysis by upregulating key glycolytic genes, while simultaneously inhibiting oxidative phosphorylation (92, 93). Beyond glucose metabolism, HIF-1α regulates the uptake and utilization of amino acids, fatty acids, and choline, thereby enabling tumor cells to sustain growth under metabolic stress. This broad influence extends to immune and stromal cells within the tumor microenvironment, altering nutrient availability and metabolic signaling in ways that further complicate treatment response (94). Hypoxia can profoundly reshape tumor cell metabolism and influence therapeutic response. In solid tumors, hypoxic conditions are a major driver of resistance to radiotherapy, a phenomenon closely associated with HIF-1α. HIF-1α activation under low-oxygen conditions promotes adaptive metabolic reprogramming, enabling cancer cells to survive and proliferate despite metabolic stress. In breast cancer, hypoxia-induced HIF-1α signaling has been strongly implicated in radioresistance, partly through its effects on glucose metabolism. Pharmacological interventions targeting this pathway have shown promise; for example, chrysin in combination with radiotherapy suppresses radiation-induced HIF-1α expression, thereby enhancing the radiosensitivity of TNBC cells (95). The role of HIF-1α in cancer radioresistance has been comprehensively reviewed by Harada et al. (96). Here, we highlight the central role of HIF-1α in modulating breast cancer radiosensitivity through the regulation of glucose metabolism reprogramming.

Recent findings emphasize that HIF-1α-driven glycolytic reprogramming contributes to mitochondrial dysfunction and T cell exhaustion. Inhibition of this process helps maintain stemness, persistence, and cytotoxic activity of chimeric antigen receptor (CAR) T cells, which is crucial for durable immunotherapy efficacy (97). At the molecular level, miR-210-3p has been shown to stabilize HIF-1α by targeting glycerol-3-phosphate dehydrogenase 1-like (GPD1L), while simultaneously suppressing p53 activity through cytoglobin (CYGB). This dual regulation facilitates aerobic glycolysis by coordinating downstream targets of both HIF-1α and p53. Additionally, mTORC1 promotes HIF-1α translation, further amplifying glycolytic enzyme expression (98). Through these mechanisms, HIF-1α enhances glycolysis, autophagy, and angiogenesis, collectively fostering radioresistance in TNBC.

### 3.3 P53 and PTEN

The tumor suppressor p53 integrates diverse stress signals, including hypoxia, DNA damage, and excessive proliferative stimuli, to orchestrate cellular responses such as cell-cycle arrest, DNA repair, metabolism, angiogenesis, and apoptosis. p53 activity is regulated by upstream kinases including ATM and ATR, as well as negative regulators such as MDM2 and MDMX. Notably, p53 is the most frequently mutated gene in TNBC, with mutations occurring in up to 80% of cases (99). Such mutations abrogate its tumor-suppressive functions and facilitate metabolic reprogramming that supports cancer progression. Multiple therapeutic strategies targeting P53 have been developed and are either in clinical use or are undergoing preclinical and clinical evaluation for treatment of TNBC (100). In addition, several natural compounds have been shown to modulate radiosensitivity in TNBC by regulating P53 expression. For example, recent studies reported that green Ag-NPs synthesized from pumpkin peel extract can enhance p53 expression by inhibiting HIF-1α, thereby increasing the radiosensitivity of TNBC cells (101).

PTEN is another tumor suppressor frequently altered in TNBC, with mutation or deletion rates second only to those of P53 (102). As a negative regulator of the PI3K–AKT signaling pathway, PTEN plays a critical role in controlling glucose metabolism in tumor cells (103). A high frequency of PTEN deletion is closely associated with early recurrence, larger tumor size, and higher pathological grade in TNBC patients (104, 105), underscoring its importance in disease progression. Elevated glucose levels can induce PTEN ubiquitination, promote breast cancer cell proliferation, and reduce the therapeutic efficacy of the NEDD8-activating enzyme inhibitor MLN4924 (pevonedistat) (106). Conversely, PTEN overexpression enhances TNBC sensitivity to 5-fluorouracil by suppressing glycolytic capacity (107). Notably, concurrent inactivation of PTEN and TP53 represents the most common genetic alteration pattern in invasive basal-like breast cancer, with the majority of PTEN-deficient tumors also exhibiting p53 loss. In this context, PI3Kβ has been shown to drive the formation of an immunosuppressive tumor microenvironment. Combined treatment with PI3Kβ inhibitors and PD-1 blockade synergistically suppresses tumor growth in PTEN- and p53-deficient breast cancers (108). These findings indicate that PTEN and p53 jointly influence the sensitivity of breast cancer cells to radiotherapy and chemotherapy by modulating glucose metabolism reprogramming.

Beyond these key regulators, additional factors contribute to TNBC progression and treatment response through metabolic remodeling. Colony-stimulating factor 3 (CSF3) is markedly enriched in the secretome of hypoxia-activated cancer-associated fibroblasts and promotes glycolysis in TNBC cells by activating phosphoglucomutase 2-like 1 (PGM2L1) downstream of its receptor CSF3R, thereby supporting malignant phenotypes (109). N-acetyltransferase 10 (NAT10) has also been identified as a promoter of TNBC progression. Genetic deletion or pharmacological inhibition of NAT10 suppresses tumor growth and enhances T-cell activation, while combination therapy with a NAT10 inhibitor and CTLA-4 monoclonal antibody further improves antitumor immunity. Mechanistically, NAT10 regulates glycolysis and immune suppression through the NAT10–ac4C–JunB–LDHA signaling axis (110). Additionally, long non-coding RNAs participate in metabolic regulation; LINC02159 has been shown to promote glycolysis and proliferation in TNBC cells by modulating the miR-1285-3p/G6PI axis (111).

Collectively, these findings highlight glucose metabolism reprogramming as a central determinant of radiotherapy efficacy in TNBC. The regulatory network governing tumor metabolism is highly complex and involves multiple genetic, epigenetic, and microenvironmental factors. Further mechanistic and translational studies are required to fully elucidate these pathways and to develop more effective metabolism-based therapeutic strategies for TNBC.

## 4 How glycolysis influences TNBC radiotherapy sensitivity

The altered metabolism in cancer cells offers a way to develop cancer cell - specific therapeutic targets and anti-cancer agents. In fact, therapeutic strategies targeting glycolysis and cancer cell- specific biosynthetic pathways are a major focus in cancer research. Comparison of radiosensitized and radiation-resistant cancer cells showed that radiation-resistant cells had higher rates of glycolysis, increased glucose uptake, and more lactate production (112). Lin et al. (113) confirmed that glycolytic activity can serve as a promising indicator for predicting the efficacy of radiotherapy in breast cancer patients. Glycolysis, as a central component of reprogrammed glucose metabolism, is tightly linked to the progression and therapeutic resistance of cancer cells. Aberrant glucose utilization and heightened glycolytic flux have been associated with poor radiotherapy response, reflecting the critical role of metabolic plasticity in treatment failure. Aerobic glycolysis not only fuels tumor growth but also reshapes the immune microenvironment, creating conditions in which TNBC cells evade immune attack and resist radiotherapy or drug therapy. By reprogramming their metabolic pathways, TNBC cells maintain robust energy production and biosynthesis to survive under microenvironmental stress. An important hallmark of cancer progression is metabolic reprogramming, which occurs not only in tumor cells but also in immune cells within the tumor microenvironment (114). For instance, endothelial lipase has been shown to mediate histone deacetylase 6 (HDAC6) activity and histone acetylation, altering the expression of interleukin-6 (IL-6) and fatty acid synthesis genes, thereby modulating oxidative metabolism in TNBC cells (115). Although immune checkpoint blockade (ICB) therapy has shown promise in TNBC, the proportion of responsive patients remains relatively low. To overcome this limitation, combining radiotherapy with immunotherapy has emerged as a synergistic approach. Emerging evidence indicates that metabolic modulation can enhance both immunotherapy and radiotherapy in TNBC. For example, D-mannose has been shown to promote therapeutic efficacy by facilitating PD-L1 degradation (116). In preclinical models, ^177^Lu-DNP-DOTA-BSA combined with ICB significantly enhanced therapeutic efficacy and prolonged survival in TNBC-bearing mice, although clinical validation is still lacking (117). In addition, Huang et al. (118) demonstrated that the CD155/TIGIT signaling suppresses glucose metabolism in CD8^+^ T cells through inhibition of the PI3K/AKT/mTOR pathway, thereby creating an immunosuppressive metabolic environment that favors TNBC progression. Collectively, these findings suggest that TNBC cells can remodel the immune microenvironment through metabolic reprogramming, and they highlight the therapeutic potential of strategies that simultaneously target immune suppression and tumor metabolism.

Accumulating evidence highlights the therapeutic relevance of glycolytic pathway genes in TNBC, many of which exhibit prognostic value independent of clinical variables. Key glycolysis-related genes, including HK2, LDHA, PFKP, PGAM1, GPI, SLC2A6, and ENO1, have been identified as predictors of patient outcomes. Among these, the transcription factor Y-box binding protein 1 (YBX1) has been strongly associated with glycolysis-related gene expression signatures and is overexpressed in TNBC, suggesting its dual role in regulating glycolysis and driving epithelial–mesenchymal transition (EMT) (119). Moreover, TNBC cells have been shown to depend on dihydrolipoamide S-succinyltransferase (DLST), a key enzyme in the TCA cycle. Targeting DLST with the lipoate analog CPI-613 reduced tumor burden and invasiveness, underscoring the therapeutic potential of interfering with TCA cycle metabolism (120). Collectively, these findings demonstrate that metabolic reprogramming, particularly involving glycolysis and its related pathways, plays a critical role in determining chemoradiotherapy sensitivity in TNBC. Targeting metabolic enzymes, signaling cascades, and regulators such as HIF-1α offers promising avenues for enhancing radiosensitivity.

**Figure 1:**
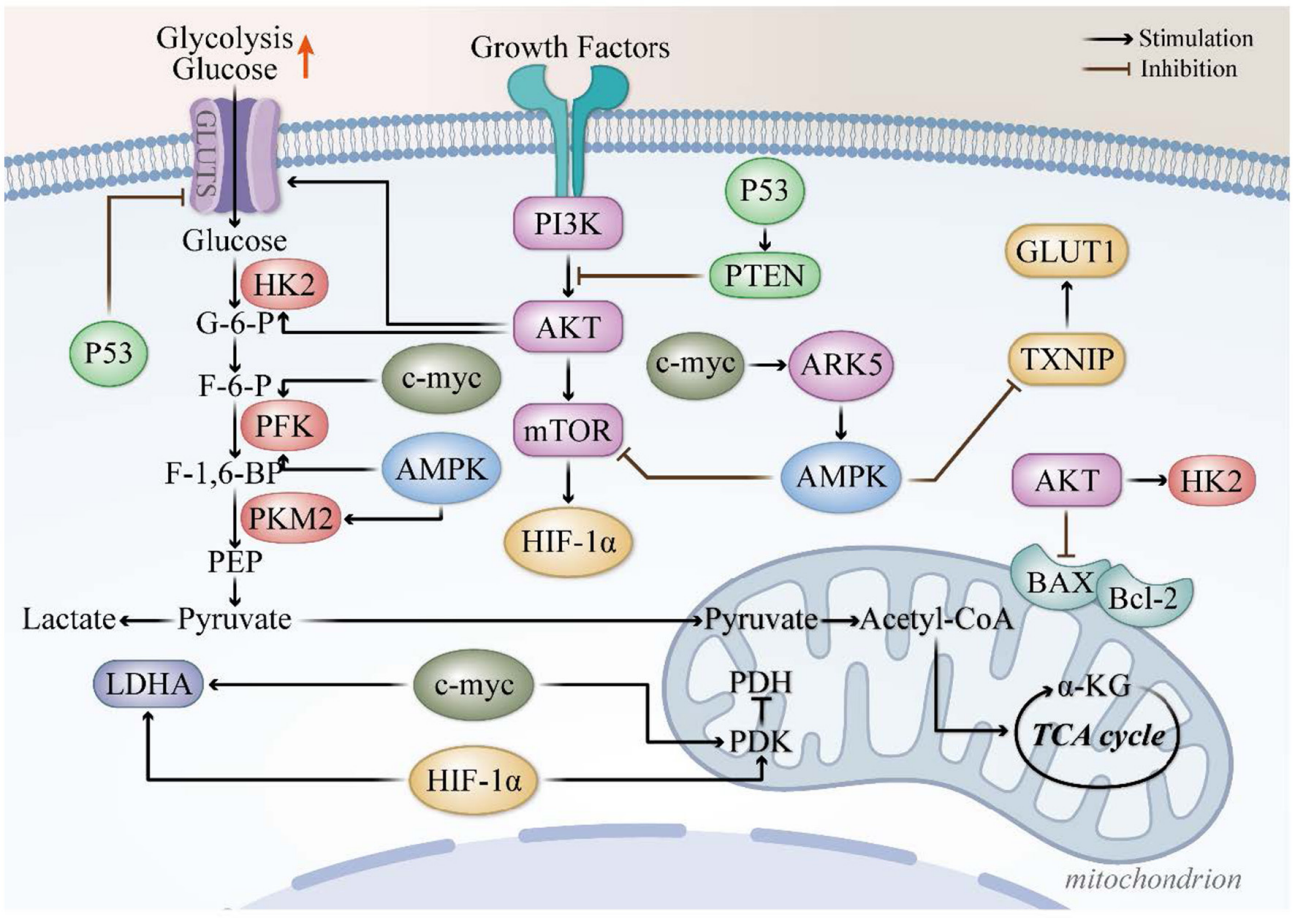
Possible pathway for improving the sensitivity of TNBC radiotherapy through reprogramming of glycolytic metabolism. The diagram illustrates the key components and interactions between glucose metabolism and radiotherapy target gene, including AMPK [Adenosine 5′-monophosphate (AMP)-activated protein kinase], c-myc (c-myc proto-oncogene), GLUT1 (glucose transporter type 1), HIF-1α (hypoxia-inducible factor 1-alpha), mTOR (Mechanistic target of rapamycin), PI3K (phosphoinositol-3 kinase), AKT (protein kinase B), PKM2 (pyruvate kinase M2), G-6-P (glucose-6-phosphate), F-6-P (fructose-6-phosphate), F-1,6-P (fructose-1,6-bisphosphate), PEP (phosphoenolpyruvate), LDHA (lactate dehydrogenase A); and TCA cycle (tricarboxylic acid cycle).

Targeting metabolic or survival pathways can impair DNA damage repair and enhance radiation-induced apoptosis, thereby increasing radiosensitivity. AMPK, a cellular energy sensor, is activated under low-energy conditions and is further induced by radiotherapy. Both AMPKα1 and AMPKα2 subunits are widely expressed in TNBC. Knockdown of AMPKα1 in the MDA-MB-231 TNBC cell line induces cell-cycle arrest, upregulates p53 expression, and suppresses glycolytic activity. Notably, radiotherapy has been shown to increase both AMPKα activation and total protein levels in these cells (121). In parallel, the PI3K pathway, a key regulator of cellular metabolism and survival, plays a critical role in modulating radiosensitivity in breast cancer. Akt, a downstream effector of PI3K, forms a bidirectional negative feedback loop with AMPK, jointly regulating metabolic balance and stress responses in TNBC cells (122). Inhibition of Akt1 enhances radiosensitivity, further supporting the involvement of the PI3K-AKT-mTOR axis in radiotherapy response (123). Based on these findings, we propose a mechanistic framework in which modulation of glycolytic metabolism through AMPK and PI3K–Akt–mTOR signaling may improve TNBC radiosensitivity, as summarized in the accompanying schematic (Figure 1).

## 5 Outlook

The high incidence and mortality of TNBC continue to pose a serious threat to women's health worldwide. While postoperative radiotherapy has markedly improved patient survival, the inherent or acquired radioresistance of TNBC cells often undermines therapeutic efficacy. Overcoming this challenge by identifying molecular targets that enhance radiosensitivity holds significant clinical value for improving treatment outcomes. In recent years, abnormal energy metabolism has attracted increasing attention as both a hallmark of TNBC and a promising avenue for therapeutic intervention. As the metabolic foundation of TNBC progression, glucose metabolism reprogramming is emerging as a critical determinant of therapeutic resistance and thus a potential breakthrough point for treatment innovation.

Through metabolic reprogramming, TNBC cells acquire energy and biosynthetic precursors that fuel proliferation, invasion, and immune evasion, largely mediated by high levels of glucose metabolism–related enzymes. Consequently, targeting key glycolytic enzymes or their regulatory pathways represents an appealing therapeutic strategy. Recent advances have led to the development of small-molecule inhibitors and natural compounds aimed at metabolic enzymes, with encouraging preclinical results. However, challenges such as limited half-life, poor solubility, and potential toxicities have restricted their clinical translation. Moving forward, a more personalized approach will be essential. Strategies such as molecular screening and biomarker-guided selection of metabolic targets may enable the design of individualized therapies, potentially reducing the required number of radiotherapy sessions, shortening treatment cycles, and improving efficacy.

Nevertheless, the metabolic complexity and heterogeneity of TNBC underscore the need for extensive experimental and translational research. A deeper understanding of the mechanisms underlying glucose metabolic reprogramming, and its contribution to radioresistance, will be crucial to identify clinically viable targets. Ultimately, integrating metabolic therapy with radiotherapy has the potential to overcome TNBC's resistance, opening new avenues to improve patient outcomes.
